# Stem cell therapy for heart failure in the clinics: new perspectives in the era of precision medicine and artificial intelligence

**DOI:** 10.3389/fphys.2023.1344885

**Published:** 2024-01-09

**Authors:** Mohammed A. Chowdhury, Jing J. Zhang, Rodrigue Rizk, William C. W. Chen

**Affiliations:** ^1^ Division of Basic Biomedical Sciences, Sanford School of Medicine, University of South Dakota, Vermillion, SD, United States; ^2^ Department of Public Health and Health Sciences, Health Sciences Ph.D. Program, School of Health Sciences, University of South Dakota, Vermillion, SD, United States; ^3^ Department of Cardiology, North Central Heart, Avera Heart Hospital, Sioux Falls, SD, United States; ^4^ Department of Computer Science, University of South Dakota, Vermillion, SD, United States

**Keywords:** stem cells, heart failure, cell therapy, precision medicine, artificial intelligence, machine learning, clinical trial, regenerative medicine

## Abstract

Stem/progenitor cells have been widely evaluated as a promising therapeutic option for heart failure (HF). Numerous clinical trials with stem/progenitor cell-based therapy (SCT) for HF have demonstrated encouraging results, but not without limitations or discrepancies. Recent technological advancements in multiomics, bioinformatics, precision medicine, artificial intelligence (AI), and machine learning (ML) provide new approaches and insights for stem cell research and therapeutic development. Integration of these new technologies into stem/progenitor cell therapy for HF may help address: 1) the technical challenges to obtain reliable and high-quality therapeutic precursor cells, 2) the discrepancies between preclinical and clinical studies, and 3) the personalized selection of optimal therapeutic cell types/populations for individual patients in the context of precision medicine. This review summarizes the current status of SCT for HF in clinics and provides new perspectives on the development of computation-aided SCT in the era of precision medicine and AI/ML.

## Introduction

Heart failure (HF) typically arises from prolonged cardiomyopathy, a chronic and progressive pathological condition characterized by weakening, loss, and/or stiffening of the heart muscle (i.e., myocardium) (Dassanayaka and Jones, 2015). Without proper intervention, cumulative reductions in the cardiac capacity to pump blood likely lead to HF or even death. Unfortunately, HF is irreversible and incurable because human hearts do not have sufficient innate regenerative capacity to restore severe myocardial damage (Uygur and Lee Richard, 2016). HF has become a major global healthcare burden that progressively deteriorates the physiological capability of the affected population and significantly impacts their quality of life (Savarese et al., 2022). In the United States alone, HF affects around 2.5% and 1.7% of all men and women, respectively (Tsao et al., 2022). Importantly, the overall age-adjusted mortality rate for HF has notably increased from 2.36 to 3.16 per 100,000 people over the recent two decades (1999–2019) (Jain et al., 2022).

The current medical regimen for clinical Stage C symptomatic HF includes a combination of vasodilators, beta blockers, sodium-glucose cotransporter-2 inhibitors, mineralocorticoid receptor antagonists, and diuretics (Heidenreich et al., 2022). Regardless of the recommended medical treatment, about 5% of HF patients develop Stage D HF or end-stage heart disease that requires either heart transplantation or mechanical support with a left ventricular (LV) assist device (LVAD) (Costanzo et al., 2008). However, these advanced therapies for end-stage HF have their individual limitations. For example, there is a constant shortage of matching hearts for transplantation as well as a higher incidence of sudden cardiac death in heart transplant recipients compared to the general population (Colvin et al., 2022; Bonnet et al., 2023). Patients with LVAD are at an increased risk for thromboembolic complications, bleeding, driveline infection, and right ventricular failure (Chaudhry et al., 2022). Thus, there is an unmet need for alternative medical approaches that fundamentally stop or revert the progression of HF pathologies as well as biologically enable the preservation and/or regrowth of functional myocardium.

**FIGURE 1:**
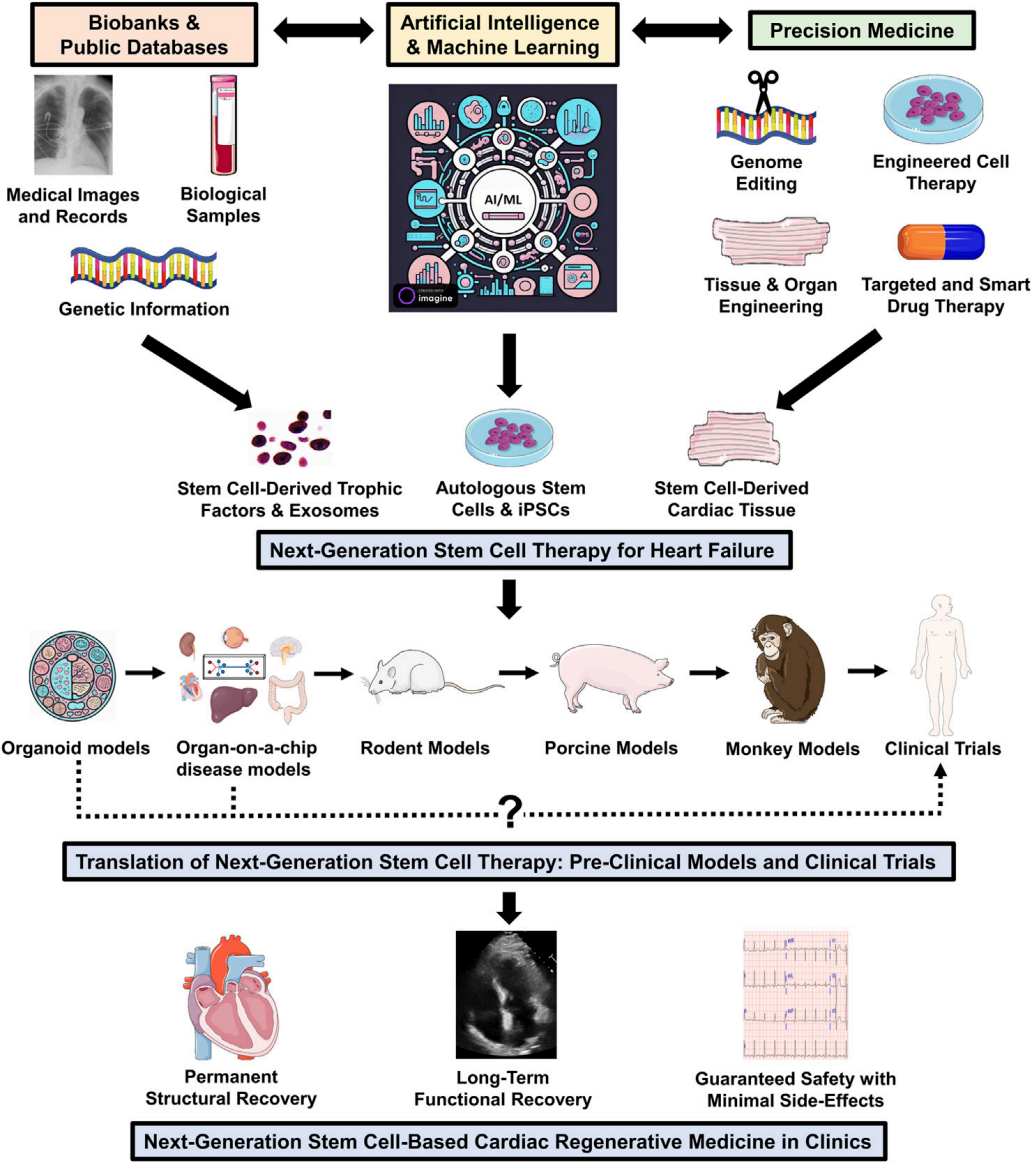
Next-generation stem/progenitor cell therapy for heart failure. To design next-generation personalized stem cell treatment for HF that ensures sustainable functional and structural recovery with minimal side effects, it is essential to integrate new components, including AI/ML, bioinformatics, and precision medicine, into stem cell research and therapeutic development. The emerging streamlined high-throughput testing platforms, such as organoid and organ-on-a-chip disease models, may greatly shorten the preclinical development phase and accelerate the progress of human trials. The Figure was partly generated using Servier Medical Art, provided by Servier, licensed under a Creative Commons Attribution 3.0 unported license.

Stem cells are precursor cells that have the ability to self-renew and differentiate into functionally mature, specialized cells in various human tissues (i.e., pluripotent or multipotent) (Evans and Kaufman, 1981; Pittenger et al., 2019). Numerous efforts have been poured into stem cell research over the last two decades, resulting in abundant laboratory discoveries and translational applications of distinct human stem/progenitor cell types: embryonic stem cells (ESCs), induced pluripotent stem cells (iPSCs), lineage-restricted or tissue-specific stem/progenitor cells (e.g., hematopoietic stem cells, skeletal muscle satellite cells, and intestinal stem cells) (Martello and Smith, 2014; Pizzute et al., 2015; Moradi et al., 2019), and adult mesodermal multipotent precursor cells (e.g., mesenchymal stem/stromal cells) (Pittenger et al., 2019). Many human clinical trials using stem cell-based regenerative therapy for treating HF have thus arisen from promising basic stem cell research and demonstrated encouraging results (Table 1). (Hare et al., 2012; Perin et al., 2012; Heldman et al., 2014; Hare et al., 2017; Teerlink et al., 2017) In this review, we will summarize the current status of stem/progenitor cell therapy for HF, persistent challenges and possible solutions, as well as the future perspectives of stem cell-based cardiac regenerative medicine in the era of precision medicine and artificial intelligence (AI) (Figure 1).

**TABLE 1:** Summary of recent clinical trials with stem cell therapy for heart failure. This table summarizes the key parameters and findings of major human trials with stem/progenitor cell therapy for heart failure since 2015.

Trial Name Author Year of Publication Trial Phase	Administration Route and Type of Stem Cells	Type of HF (Pt #)	Randomization and Sample Size	Average Age (% Male)	Average EF	NYHS Class	Key Findings
REGENERATE-DCM Hamshere et al. (2015) Phase II	Intracoronary administration of autologous BMC	NICM	S/C Saline:15 S/C G-CSF:15 IC BM serum:15 IC BMC: 15	56 (63%)	36%	≥II	At 3 months post-treatment, the IC BMC therapy group showing 1) 5.37% increase in LVEF: 38.3% ± 13.0% vs. 32.9% ± 16.5% (*p* = 0.0138) for up to 1 year. 2) Decrease in NYHA classification, reduced plasma NT-proBNP,increased exercise capacity, and improved quality of life. 3) No notable change in LVEF in remaining intervention groups
MiHeart Study Martino et al. (2015) Phase II/II	Intracoronary administration of autologous BMNC	NICM	Placebo: 78 IC BMNC: 82	56 (73%)	24%	III/IV	At 12 months post-treatment, no significant differences between the intervention and placebo groups for LVEF, LVESV, LVEDV, and mortality rate
MPC-HF Perin et al. (2015) Phase II	Transendocardial administration of allogenic MSC	ISCM(38) and NICM (7)	25M MPC: 20 75M MPC: 20 150M MPC: 20 (15 treated and 5 mock control 5 mock control each per group)	62 (97%)	31%	II/III	1) No difference between the groups for adverse events, clinically significant immune response, survival probability, MACE-free probability, and all-cause mortality. 2) Significant reduction in HF-related MACE (HF hospitalization, successfully resuscitated cardiac death, or cardiac death) in the 150M MPC group compared to all other groups (*p* = 0.025)
MSC-HF trial Mathiasen et al. (2020) Phase II	Intracoronary administration of autologous BM derived MSC	ISCM	Placebo: 20 MSC: 40	66 (36%)	28%	II/III	At 3 months post-treatment, the MSC therapy group showing: 1) Significant reduction in LVESV (−7.6 mL, *p* = 0.001). 2) No significant change in LVEF, stroke volume, and myocardial mass
REGENERATE-AMI Choudry et al. (2016) Phase II	Intracoronary administration of allogenic BMC	ISCM	BMC: 55 Placebo: 45	56 (84%)	48%	≥I	1) At 1 year, a greater myocardial salvage index by MRI in the BMC-treated group, compared with placebo (*p* = 0.048). 2) No difference in rare major adverse events between groups. 3) At the 5-year follow-up, there was no difference in the clinical outcomes between the two groups. Mathur et al. (2022)
IMPACT-CABG Noiseux et al. (2016) Phase II	Intramyocardial administration of autologous BM derived CD133+ Cells	ISCM	Intervention: 19 Placebo: 14	66 (89.5%)	40%	II-IV	1) At 6 months post-treatment, improvements in LVEF and LV volumes in all patients by MRI with no significant difference between the two groups. 2) One death and four cases of transient renal insufficiency during the 6-month follow-up period
Ixmyelocel-T for ISCM Patel et al. (2016) Phase IIB	Intramyocardial administration of autologous Ixmyelocel-T (BM deribed-CD90^+^ MSC and Cd 45+CD14+macrophages	ISCM	Ixmyelocel-T: 66 Placebo: 60		35%	III/IV	At 12 months post-treatment, the Ixmyelocel-T therapy comparing to the placebo group: 37% reduction in cardiac events (risk ratio: 0.63 [95% CI 0.42– 0.97]; *p* = 0.0344). Less serious adverse events (53% vs 75%, *p* = 0.0197)
CHART-1 Study Teerlink et al. (2017) Phase II/III	Intramyocardial administration of autologous cardiopoietic MSC	ISCM	Cardiopoietic MSC (C3BS- CQR-1): 120 Sham procedure: 151	62 (89%)	27%	II-IV	1) At 12 months post-treatment, the cardiopoietic MSC group showing decreases in LVEDV by 17 mL (*p* = 0.006) and increases in LVESV by 12 mL (*p* = 0.017). 2)The treatment group with a moderate number of repeated injections (>16 to <20) exhibiting the largest reverse remodeling
PERFECT Steinhoff et al. (2017) Phase III	Intramyocardial administration of autologous BM derived CD133+ stem cells	ISCM	CD133+ SC: 41 Placebo: 41	63 (85%)	32%	I-IV	1) At 180 days post-treatment, no notable difference in survival, adverse events, or change in LVEF by MRI from baseline. 2) Increased Erythropoeitin (*p* = 0.02) and SH2B3 mRNA expression (*p* = 0.073) in preoperative peripheral blood of the responders (∆LVEF≥ 5% after 180 days); reduced CD133+ EPC (*p* = 0.005) and thrombocytes (*p* = 0.004) in the preoperative peripheral blood of the non-responders. 3) Preoperative discrimination with 80% (responders) and 84% (non-responders) accuracy after 10-fold cross-validation by machine learning-identified 20 biomarker response parameters
REGENERATE-IHD Choudhury et al. (2017) Phase II	Intramyocardial and intracoronary administration of autologous BMSC with G- CSF	ISCM	Peripheral: S/C G-CSF: 15 S/C Placebo: 15 Intramyocardial: IM BMC: 15 S/C Placebo: 15 Intracoronoary: IC BMC: 15 IC Placebo: 15	61 (100%)	30%	II-IV	1) At 1 year post-treatment, significant improvement in LVEF of 4.99% by MRI with intramyocardial BMC therapy (*p* = 0.038); no difference in LVEF in all other groups. 2) Reduced NT-proBNP at 6 months and a reduction in NYHA class at 1 year with intramyocardial BMC therapy
Muscle-derived SC with connexin-43 gene overexpression for HF Gwizdala et al. (2017) Phase I	Intramyocardial administration of allogenic engineered muscle derived stem/progentor cells	ISCM (11) and NICM (2)	13	61 (92%)	25%	II/III	At 6 months, compared to the baseline: 1) Improved exercise capacity: NYHA (3 ± 0 vs. 1.8 ± 0.7, *p* = 0.003), exercise duration (388.7 ± 141.8 s vs. 462.1 ± 176.7 s, *p* = 0.025), peak O2 consumption (14.4 ± 4.0 vs. 15.8 ± 3.7 mL/kg.min, *p* = 0.022), and O2 pulse (10.6 ± 2.9 vs. 18.9 ± 22.6 mLO2/heart rate, *p* = 0.012). 2) Improvement in the levels of BNP, LVEF, and LVED. 3) Significant improvement in the mean unipolar voltage amplitudes in the injected segments (9.6 ± 2.6 vs. 11.6 ± 3.5 mV, *p* = 0.014) and the entire LV (8.8 ± 2.8 vs. 10.2 ± 3.4mV, *p* = 0.041). 4) No deaths reported; one subject with ventricular tachycardia
TRIDENT study Florea et al. (2017) Phase II	Transendocardial administration of allogenic BM derived MSC	ISCM	20M-MSC: 15 100M-MSC: 15	66 (90%)	36%	I-III	At 12 months post-treatment: 1) Similar reduction in scar size in both groups by CT. 2) Increase in LVEF only with 100M-MSC Tx (by 3.7U, *p* = 0.04).3) Improved NYHA class in the 20M-MSC (35.7%) and 100M-MSC (42.9%) groups 4) Increased proBNP in the 20M-MSC group (0.32 log pg/mL *p* = 0.039), but not in the 100M-MSC group (−0.07 log pg/mL)
RIMECARD Trial Bartolucci et al. (2017) Phase I/II	Peripheral infusion of allogenic umbilical cord derived MSC	ISCM (21) and NICM (9)	UC-MSC: 15 Placebo: 15	57 (80%)	33%	I-III	At 3, 6, and 12 months post-treatment, the US-MSC group had: significant improvement in LVEF compared to baseline (+7.07 ± 6.22% vs. +1.85 ± 5.60%; *p* = 0.028) improvements of NYHA class (*p* = 0.0167 vs. baseline) and MLHFQ (*p*<0.05 vs. baseline). no difference in mortality, HF admissions, arrhythmias, or incident malignancy between the two groups
IV-MSC for NICMP Butler et al. (2017) Phase IIA	Peripheral infusion of allogenic ischemia-tolerant MSC (itMSC) grown in chronic hypoxia	NICM	itMSC: 10 Placebo: 12	47 (59%)	32%	II/III	No difference in mortality, adverse events, or hospitalization. No significant change in LVEF and LV volume. Increased 6-min walk distance (+36.47 m, 95% CI 5.98– 66.97; *p* = 0.02) with itMSC Tx. 3) Improved Kansas City Cardiomyopathy clinical summary (+5.22, 95% CI 0.70–9.74; *p* = 0.02) and functional status scores (+5.65, 95% CI −0.11 to 11.41; *p* = 0.06) with itMSC Tx
IC BMC and MSC in HF Xiao et al. (2017) Phase II	Intracoronary administration of autologous BM mononuclear cells or MSC	NICM	BMMC: 16 BMSC: 17 Control: 20	50 (64%)	33%	II-IV	At 3 months, improvement in LVEF (*p* = 0.004), NYHA class (*p* = 0.02) and myocardial perfusion (*p* = 0.019) with BMSC Tx as well as LVEF (*p* = 0.04) and NYHA class (*p* = 0.047) with BMMC Tx. At 12 months, improvement in LVEF (*p* = 0.005), NYHA class (*p* = 0.05) and myocardial perfusion (*p* = 0.038) only with BMSC Tx. No difference in major adverse cardiovascular events between the three groups
POSEIDON-DCM Hare et al. (2017) Phase I/II	Transendocardial administration of allogenic or autologous BM derived MSC	NICM	Autologous BMSC: 19 Allogenic BMSC: 18	56 (71%)	26%	I-III	At 1 year post-treatment: 1) Increase in LVEF in allo-BMSC group by 8.0% (*p* = 0.004) compared with auto-BMSC; 2) Increase in the 6-min walk test with allo-BMSC by 37.0 m (*p* = 0.04); 3) Decrease in MLHFQ score in allo-BMSC (*p* = 0.0022); 4) Decreases in TNFα overall for both groups (*p* = 0.0001) with a greater decrease in the allo-BMSC group (*p* = 0.05); 5) No serious adverse events at 30 days; at12 months, serious adverse event rates: 63.5% in auto-BMSC and 28.2% in allo-BMSC (*p* = 0.1)
Repeat CD34^+^ Vrtovec et al. (2018) Phase II	Transendocardial administration of autologous peripheral blood stem cells	NICM	Group A: Repeated stem cell treatment in 6 months: 30 Group B: Single stem cell treatment: 30	55 (88%)	31%	III	1) From baseline to 6 months, improvement in both groups: a. LVEF: +6.9 ± 3.3% in Group A, *p* = 0.001 and +7.1 ± 3.5% in Group B, *p* = 0.001. b. NT-proBNP: −578 ± 211 pg/mLin Group A, *p* = 0.02 and −633 ± 305 pg/mL in Group B, *p* = 0.01. c. 6-min walk test: +87 ± 21 m in Group A, *p* = 0.03 and +92 ± 25 m in Group B, *p* = 0.02)
RECARDIO Bassetti et al. (2018) Phase I	Intramyocardial administration of autologous BM derived CD133+ cells	ISCM	10	69 (100%)	38%	II-IV	1) At 6 months, improved baseline myocardial perfusion in: Summed stress scores (from 18.2 ± 8.6 to 13.8 ± 7.8, *p* = 0.05). Difference stress scores (from 12.0 ± 5.3 to 6.1 ± 4.0, *p* = 0.02). Improvement at 6 months compared to baseline in Canadian Cardiovascular Society (*p*≤0.001) and NYHA classes (*p* = 0.007). Positive correlation between changes in summed stress score and ATMP-CD133 release of proangiogenic cytokines HGF (*r* = 0.80, *p* = 0.009) and PDGF-bb (*r* = 0.77, *p* = 0.01). Negative correlation between changes in summed stress score and the proinflammatory cytokines RANTES (*r* = −0.79, *p* = 0.01) and IL-6 (*r* = −0.76, *p* = 0.02)
MPC in LVAD Yau et al. (2019) Phase II	Intramyocardial administration of allogenic MPC	ISCM (70) and NICM (89)	MPC: 106 Control: 53	55 (88.7%)	15%	II-IV	No difference between the groups in terms of successful temporary weaning from LVAD after 6 months of randomization, rate of adverse events, rate of readmission, and 1-year mortality
HUC-HEART Trial Ulus et al. (2020) Phase I/II	Intramyocardial administration of allogenic umbilical derived MSC vs BM mononuclear cells	ISCM	Control: 16 BM-MNC: 12 Umbilical MSC: 25	59 (100%)	35%	I/II	At the 6-month follow-up: decline in NT-proBNP levels compared to baseline in both cell-treated groups. At the 6- to 12-month follow-up: increase in LVEF (5.4%) and stroke volume (19.7%) only in the umbilical MSC group. Decreasing necrotic myocardium by 2.3% in the control, 4.5% in the BM-MNC group, and 7.7% in the umbilical MSC group. Increase in the 6-min walking test in the control (14.4%) and the umbilical MSC group (23.1%)
CCTRN SENECA Trial Bolli et al. (2020) Phase I	Intramyocardial administration of allogenic BM derived MSC	NICM	MSC: 14 Placebo: 17	54 (24%)	33%	II/III	No significant difference in clinical outcomes between the two groups
Collagen scaffold MSC in HF He et al. (2020) Phase I	Intramyocardial administration of allogenic MSC with cell-laden hydrogel scaffold	ISCM	CABG + Cell + Hy drogel: 18 CABG + Cell: 17 Control: 15	62 (78%)	<10%	III/IV	No significant difference in serious adverse events. At 12 months post-treatment, cardiac MRI showing significant reduction in the mean infarct size only in the collagen/cell group: −3.1% (95% CI, −6.20% to −0.02%, *p* = 0.05)
ALLSTAR Makkar et al. (2020) Phase II	Intracoronary administration of allogenic cardiosphere derived cells	ISCM	CDC: 90 Placebo: 44	55 (84.4%)	40%	I/II	A1 month post-treatment, no primary safety endpoint events. At 6-month follow-up, no change in scar size percentage. At 6-month follow-up, CDC-treated patients showing notable reductions in LVEDV (*p* = 0.02), LVESV (*p* = 0.02), and NT-proBNP (*p* = 0.02)
CCTRN CONCERT-HF Bolli et al. (2021) Phase II	Intracoronary administration of autologous bone marrow derived MSC and c-kit + CPC	ISCM	MSC + CPC: 33 MSC: 29 CPC: 31 Placebo: 32	62 (87%)	28%	II/III	Lowest HF-related major adverse cardiac events in the CPC- treated group compared to placebo (−22%, *p* = 0.043) Significantly improved QOL scores in the MSC-alone group (*p* = 0.05) and the MSC + CPC group (*p* = 0.023) vs. placebo. No significant difference among groups in LVEF, LV volumes, scar size, 6-min walking distance, and peak O2 consumption
Danish Trial Qayyum et al. (2023a) Phase II	Intramyocardial administration of allogenic adipose derived MSC	ISCM	ASC: 54 Placebo: 27	67 (81%)	34%	II	No significant change in LVESV, LVEDV, LVEF, NYHA class and 6 min walk test between groups
SCIENCE Trial Qayyum et al. (2023b) Phase II	Intramyocardial administration of allogenic adipose derived MSC	ISCM	ASC: 90 Placebo: 43	66 (93%)	32%	II/III	No significant differences between groups in LVESV, LVEDV, LVEF, NYHA class, 6-min walk test, NT-proBNP, CRP, or QOL.
MPC in HF Perin et al. (2023) Phase III	Transendocardial administration of allogenic BM derived MSC	ISCM (319) and NICM (244)	BMSC: 283 Control: 282	63 (78%)	28%	II/III	At 12 months post-treatment, BMSC group vs. control group (analysis population: *n* = 537): Increasing LVEF, especially in patients with inflammation. Decrease in the risk of TTFE for MI or stroke by 58% (cause- specific HR: 0.42, 95% CI: 0.23–0.76). Red uction in the risk of TTFE for the 3-point MACE by: 28% (HR: 0.72, 95% CI: 0.51–1.03) Reducing risks of MI/stroke and the 3-point MACE by 75% and 38%, respectively, in patients with inflammation (hsCRP≥2 mg/L)

Legend Abbreviations.

ATMP: Autologous advanced therapy medicinal product BM: bone marrow.

BMC: Bone marrow-derived cells BMNC: Bone marrow mononuclear cells CI: confidence interval.

CRP: C-reactive protein CT: Computed tomography EF: ejection fraction.

EPC: endothelial progenitor cell.

G-CSF: Granulocyte colony-stimulating factor HF: heart failure.

hsCRP: High-sensitivity C-reactive protein IC: intracoronary.

ISCM: Ischemic cardiomyopathy LV: left ventricule.

LVEF: Left ventricular ejection fraction LVESV: Left ventricular end-systolic volume LVEDV: Left ventricular end-diastolic volume M: million.

MACE: Major adverse cardiovascular events MSC: mesenchymal stem cells.

MLHFQ: Minnesota Living with Heart Failure Questionnaire MRI: magnetic resonance imaging.

NICM: Non-ischemic cardiomyopathy.

NT-proBNP: N-terminal pro-B-type natriuretic peptide Pt: Participants.

QOL: quality of life.

RANTES: regulated on activation, normal T cell expressed and secreted; also known as Chemokine (C-C motif) ligand 5 (CCL5). ProBNP: pro-B-type natriuretic peptide.

S/C: subcutaneous.

TNFα: Tumor necrosis factor-α TTFE: Time-to-first-event.

Tx: Treatment.

### The clinical scope of stem cells in heart failure

#### Bone marrow-derived stem cells

In 2001, bone marrow-derived stem cells (BMSCs) were first transplanted into animal models of ischemic cardiac injury where the donor cells were shown to produce *de novo* myocardial and vascular structures in the peri-infarcted regions of the myocardium (Jackson et al., 2001; Orlic et al., 2001). The observed benefits were largely attributed to the paracrine release of tissue trophic factors by the donor cells, for example, VEGF and HGF promoting angiogenesis and cardiomyocyte (CM) survival, respectively (Gnecchi et al., 2005; Mabotuwana et al., 2022).

Human phase 2 clinical trials, such as FOCUS CCTRN and TAC-HFT, were conducted in patients with ischemic cardiomyopathy (ICM) who received multiple transendocardial injections of bone marrow mononuclear cells (BMMCs) in the infarcted territory. However, the results failed to demonstrate any significant improvement in LV chamber size, ejection fraction (EF), or quality of life (Perin et al., 2012; Heldman et al., 2014). Similarly, the REGENERATE AMI trial studied the impact of intracoronary infusion of autologous BMMCs in patients with ICM (Choudry et al., 2016). Despite the encouraging results at the 1-year follow-up that showed significant decreases in the infarct size and improved myocardial salvage indices in the intervention group, the 5-year follow-up did not exhibit improved clinical outcomes, suggesting short-term benefits of intracoronary BMMC infusion (Mathur et al., 2022). Interestingly, analysis of pre-transplant bone marrow (BM) samples of patients who responded to autologous BMMC therapy in the FOCUS CCTRN trial showed a higher frequency of CXCR4+ and B cells and fewer monocytes/macrophages and endothelial colony-forming cells in their BM compared to non-responders (Taylor et al., 2016). Therefore, the presence of certain subset(s) of BM progenitor and/or immune cell populations may indicate the potency of donor cells for autologous BMMC therapy (Taylor et al., 2016). The CardiAMP trial utilized the abovementioned concept and screened their subject’s BM cell potency by flow cytometry prior to the enrollment (Johnston et al., 2018; Raval et al., 2021). Subjects with ICM and favorable BM cellular composition were selected for the trial and underwent BM aspiration, followed by an enrichment process to separate the nucleated cell fraction from the plasma phase using a density-tuned dual buoy column; the enriched BM aspirate was then injected into the infarcted myocardium (Raval et al., 2021). The 12-month follow-up data on 10 patients reported significant improvement in 6-min walk distances and trends towards improved NYHA class, LVEF, and quality of life (Raval et al., 2021).

#### Mesenchymal stem cells

Mesenchymal stem cells (MSCs) are allogeneic STRO-1/STRO-3+ cells, a subpopulation of stromal cells that express CD73, CD90, and CD105 and can be extracted from BM, adipose, and other tissues (Simmons and Torok-Storb, 1991; Haynesworth et al., 1992; Zuk et al., 2001; Karantalis and Hare, 2015). MSCs are adult multipotent precursor cells with great potential for cardiac repair since they can be easily isolated from autologous sources and rapidly expanded *ex vivo* (Pittenger et al., 1999; Halvorsen et al., 2000; Miura et al., 2003; Dominici et al., 2006; Chen et al., 2015; Melo et al., 2017). MSCs have been shown to improve cardiac function in multiple preclinical animal models of cardiac injury (Amado et al., 2005; Alfaro et al., 2008; Qi et al., 2008; Chen et al., 2013). Their primary mechanism of action for cardiac repair is paracrine secretion of multiplex tissue trophic factors that stimulate cellular repair and regeneration via angiogenesis, endothelization, anti-inflammation, and anti-fibrosis (Kocher et al., 2001; Chen et al., 2009; Abdalmula et al., 2017). The direct differentiation of MSCs into desired cardiac cell types, if any, did not appear to contribute significantly to the functional recovery observed in prior studies (van der Spoel TI. et al., 2011a; Guo et al., 2020).

The MSC-HF trial reported that ICM patients treated with multiple intramyocardial injections of autologous BM-derived MSCs exhibit progressive improvement in LV end-systolic volume (LVESV), EF, and myocardial mass 12 months after their initial treatment, even reducing hospitalization for angina in the MSC-treated group after 4 years (Mathiasen et al., 2020). On 1-year follow-up, the DREAM-HF trial demonstrated that single-dose transendocardial injection of allogenic BM-derived MSCs improved LVEF, LVESV, LV end-diastolic volume (LVEDV) of treated HF patients, with 12% reduction in MI or stroke risk in patients with elevated high-sensitivity CRP (≥2 mg/L) (Perin et al., 2023). These results suggest that MSC treatment can improve clinical outcomes in HF patients for up to several years, especially for those with systemic inflammation.

In contrast, human clinical trials using adipose-derived MSCs for HF treatment failed to show any significant beneficial outcomes (Qayyum et al., 2023a; Qayyum et al., 2023b). Yau *et al.* reported that intramyocardially injecting allogenic BM-derived MSCs during LVAD implantation did not improve the successful weaning from LVAD, 1-year mortality, or the rate of serious adverse events (Yau et al., 2019). Currently, the STEMVAD trial (NCT03925324) is evaluating the safety and efficacy of three serial doses of allogenic MSCs by intravenous infusions in patients with end-stage HF requiring LVAD. The results of this study will help clarify the utility of MSC therapy in patients with end-stage HF.

#### Cardiosphere-derived cells

Cardiosphere-derived cells (CDCs) are characterized by their ability to separate from cardiac tissues and form spheroids in suspension cultures (Messina et al., 2004). They can function as adult stem/progenitor cells and have been shown to differentiate into myocytes and vascular cells in SCID beige mice (Messina et al., 2004). CDCs mainly contribute to cardiac repair by releasing paracrine factors and exosomes which inhibit cellular apoptosis and promote angiogenesis and CM proliferation (Chimenti et al., 2010; Ibrahim et al., 2014). The ALLSTAR trial evaluated the safety and efficacy of intracoronary delivery of allogenic CDCs in ICM patients with >15% scar burden (Makkar et al., 2020). At the 6-month follow-up, the intervention group showed significant reductions in LVESV, LVEDV, N-terminal pro-B-type natriuretic peptide (NT-proBNP) levels, and decreased segmental circumferential strain with MRI, but no improvement in their LV scar size, suggesting that CDCs could functionally benefit such patients but are not anti-fibrotic (Ostovaneh et al., 2021).

#### Induced pluripotent stem cells

In 2006, Yamanaka and colleagues first described a cocktail of four transcription factors (Oct3/4, Sox2, c-Myc, and Klf4) capable of artificially reprogramming mouse embryonic cells and adult fibroblasts into iPSCs that exhibit the self-renewability and pluripotency similar to ESCs (Takahashi and Yamanaka, 2006). iPSCs possess multiple translational advantages over ESCs: 1) no ethical concerns regarding the cellular origin; (Zheng, 2016); 2) autologous immunocompatible cell sources (if applicable), such as patient’s own fibroblasts, obviating the need for immunosuppression; (Mandai et al., 2017; Schweitzer et al., 2020); 3) direct reprogramming approaches available for differentiating into desired tissue-specific cell types without going through the pluripotent stage, for example, direct reprogramming of human fibroblasts into CMs. (Qian et al., 2012). Cardiomyogenesis from iPSCs has been attempted previously (Yang et al., 2017; Wu et al., 2023); however, iPSC-derived CMs (iPSC-CMs) largely expressed fetal phenotypes and failed to efficiently function as adult CMs (Liao et al., 2021), limiting their clinical applicability. Recently, progress has been made to enhance the maturity of iPSC-CMs (Li et al., 2022; Hsueh et al., 2023). Besides, viral vectors used to reprogram fibroblasts to iPSCs may have the potential to cause cancer; (Okita et al., 2007) alternative non-viral delivery systems to induce iPSCs are under investigation, for example, the targeted nanoparticles (Anokye-Danso et al., 2011; Ye et al., 2016; Wang et al., 2020). iPSCs have the potential for clinical cell therapy in HF patients, and currently, two phase 1 trials are ongoing to assess the safety and efficacy of human allogenic iPSC-CMs in patients with ICM (ClinicalTrials.gov Identifier: NCT04945018 and NCT04696328).

#### Stem/progenitor cell-derived exosomes

Exosomes are extracellular vesicles that carry various proteins, lipids, and/or RNAs and play a major role in intercellular communications (Rezaie et al., 2022). Since the paracrine effect is an essential mechanism for stem/progenitor cell-mediated cardiac repair, exosomes derived from those cells that contain secretory trophic factors (e.g., pro-angiogenic and pro-survival cytokines) may constitute an alternative therapeutic approach to direct cell transplantation (Bolli et al., 2021). For instance, exosomes derived from human iPSCs had proliferative and protective effects on cardiac mesenchymal stromal cells, impacting their transcriptomic and proteomic profiles. (Bobis-Wozowicz et al., 2015). CDC-derived exosomes delivered via intramyocardial injections were shown to improve cardiac function and decrease scar size in porcine MI models (Gallet et al., 2017). A meta-analysis of ten studies using preclinical animal models of MI revealed that exosomal therapy had the potential to reduce cellular apoptosis and autophagy as well as improve cardiac function, fibrogenesis, and inflammatory response (Zheng et al., 2022).

Potential mechanisms of action of exosomal therapy in ischemic hearts include: 1) protection against myocardial reperfusion injury by reducing oxidative stress through inhibition of caspase 3/7 activation and delivery of cardioprotective microRNAs (miRs) such as miR-21 and miR-210; (Wang et al., 2015); 2) enhancement of intracellular calcium homeostasis and cardiomyocyte contraction by rescuing the expression and function of reticulum Ca2+ ATPase 2a (SERCA-2a) and ryanodine receptor 2 (RyR-2); (Li et al., 2023); and 3) improvement of cellular energy metabolism and myocardial bioenergetics without increasing the risk of arrhythmia (Gao et al., 2020). Intriguingly, besides modulating immune responses and inflammation, immune cell-derived exosomes facilitate crosstalk between immune cells and myocardial cells, which sustains ventricular function and promotes cardiac repair post-MI (Wen et al., 2021).

The use of exosomal therapy in HF patients is still under investigation, (Marbán, 2018; Duong et al., 2023), and current challenges for clinical applications include exosomal delivery, tissue targeting, and immunogenicity (Balbi and Vassalli, 2020). Moreover, exosomes may possibly carry inherent limitations or defects of their cellular origins that could impact their therapeutic efficacy (Riva et al., 2019; Andreeva et al., 2021). Thus, selecting appropriate, healthy stem/progenitor cell sources from which beneficial exosomes can be efficiently extracted is key to improving exosome-based HF therapy.

### Challenges and alternatives for discrepancy between animal and human studies

Human trials involving stem cell therapy often fail to replicate the remarkable successes in animal models of cardiac injury (Table 1). (Rheault-Henry et al., 2021; Bolli et al., 2022; Bolli and Tang, 2022) This could be attributed to multiple reasons: 1) rodent hearts may not accurately mimic the pathophysiology of human HF because they differ from human hearts in terms of size, intrinsic heart rate, (Wessels and Sedmera, 2003), and epigenomic and transcriptomic profiles; (Lin et al., 2014); 2) a number of confounding factors that can be controlled in a laboratory experiment may not be adequately controlled in a human clinical trial, leading to differences in observed outcomes (e.g., diet and genetic background); (Hasenfuss, 1998; van der Spoel et al., 2011a); 3) inconsistent results in clinical trials may be due to the variability in study protocols between different research groups/institutions in terms of donor cell types and sources, treatment dose and duration, routes of administration, and timing of stem cell therapy. (Golpanian et al., 2015).

Besides, isolating specific stem/progenitor cells out of their native niche environment could disrupt important cell-to-cell and/or microenvironmental signaling, which may lead to suboptimal therapeutic potency including reduced cell proliferation, survival, differentiation, and/or paracrine function. (Kuchina et al., 2011). Furthermore, the cardiac disease cascade in humans is complex and consists of a dynamic process of progressive tissue ischemia, hypoxia, inflammation, and/or myocardial fibrosis, making the host environment harsh for transplanted cells to survive. Another issue is inadequate cell retention and reduced cell survival after administration because only ∼11% of the delivered cells are retained in the myocardium, decreasing the overall efficiency of cell therapy (Hou et al., 2005). Currently, the process of stem/progenitor cell homing to areas of myocardial damage is not fully understood, and strategies to improve targeted cardiac delivery are under investigation (Liesveld et al., 2020).

Also, there are variabilities among patients in terms of comorbidities, risk factors, lifestyle, and genomic differences; presently, it is not clear which type of individuals will benefit most from stem cell therapy (Patel et al., 2010; Gambini et al., 2012). Additionally, the majority of human trials used LVEF, LVESV, and LVEDV as surrogates for cardiac recovery; alternative endpoints may be needed to assess the efficacy of stem/progenitor cells since multiple clinical studies have reported improvement in subjects’ quality of life and exercise tolerance without any significant increases in LVEF (Bolli et al., 2022). Thus, it may be essential to investigate alternative endpoint surrogates rather than solely relying on notable improvement in LVEF as the marker for therapeutic success (Borow et al., 2019).

There remain other challenges to solve, including 1) designing optimal cell banking strategies to maintain the therapeutic quality of donor stem/progenitor cells; 2) establishing appropriate high-throughput experimental protocols or computation algorithms to select or predict stem/progenitor cells ideal for treating a particular HF stage or pathology, respectively; and 3) building more non-invasive tools to measure how transplanted precursor cells function within the human subjects. Addressing these challenges may help improve the efficacy of stem cell therapy in human trials.

### The application of stem cells in precision medicine for heart failure

Precision medicine is the anticipated future of medicine where therapy will be tailored according to the patient’s genetic composition, environment, lifestyle, and risk factors (Ashley, 2016; Delpierre and Lefèvre, 2023). Stem cells can be used for a number of applications in precision medicine: 1) stem cell-derived CMs can be used to understand or simulate the pathophysiology of patient-specific cardiac conditions; (Musunuru et al., 2018) 2) cardiac diseases caused by genetic mutations can be replicated in patient-derived or engineered cell models with the assistance of the iPSC technology or genome editing tools, respectively; (Musunuru et al., 2018) 3) stem cell-derived cardiac cellular models can be used to test the efficacy and safety of personalized medication for individual patients; (Chen et al., 2016) and 4) autologous stem cell-derived cardiac cells may be used as a personalized therapeutic tool (Musunuru et al., 2018; Lightner and Chan, 2021).

Alternatively, cardiac organoid models derived from stem cells have been used to substitute animal and human subjects for the initial testing of the safety and/or efficacy in drug development, reducing animal and human morbidity and mortality (Azar et al., 2021). Thus, utilizing stem cells in precision medicine will not only improve our understanding of acquired cardiac disease (e.g., ICM and HF), (Bolli et al., 2022) inherent conditions (e.g., familial cardiomyopathies), (Jiang et al., 2021) and congenital heart defects (e.g., hypoplastic left heart syndrome, Ebstein anomaly, Fontan circulation with right ventricular dysfunction), (Tsilimigras et al., 2017) but also has the potential to be used as adjuvant treatment to current medical or surgical therapies.

Furthermore, large clinical datasets that comprise patient histories and characteristics, body fluid compositions, diagnostic results, tissue pathologies, imaging studies, and/or treatment effects may be used to identify, classify, or even predict the distinct signatures or behaviors of genome, epigenome, transcriptome, proteome, and/or phenome associated with a particular cardiac pathology in individual patients, and *vice versa* (Attia et al., 2019; Qiu et al., 2020; Segar et al., 2020; Liu et al., 2022). Also, many biological samples of patient-specific stem cells used in clinical trials are currently stored in biobanks (Musunuru et al., 2018; Annaratone et al., 2021). Combining the big data of multiomics with the cellular background and clinical information may facilitate a personalized multi-level analysis (Shi and Xu, 2019; Hu et al., 2020). Such comprehensive personalized analysis may improve our understanding of how stem cells behave and/or interact with other cell types under specific pathological conditions or disease stages (e.g., terminal-stage HF), ultimately aiding in the design of precision stem cell therapy for personalized medicine (Figure 1).

### Computational tools to aid future development of stem cell-based therapeutics

The fields of AI and machine learning (ML) are rapidly expanding and contributing to various medical applications, including medical imaging, personalized medicine, and robotic-assisted surgeries (Krajcer, 2022; Haug and Drazen, 2023). AI-driven decision-making is exemplified in scenarios where algorithms can process environmental and biological inputs, such as changes in the culture media, intercellular signals, or cellular behaviors, and respond accordingly based on predefined parameters (Adlung et al., 2021). For instance, AI may autonomously detect and sustain predetermined cellular phenotypes by adjusting the conditions in human stem/progenitor cell cultures (e.g., infusing specific cytokines to stimulate cell growth or adding bicarbonate to maintain consistent pH levels), keeping the culture quality and streamlining routine wet-lab tasks (Capponi and Daniels, 2023).

AI’s capability to analyze large preclinical and clinical datasets from biobanks, research data depositories, public health databases, and healthcare systems has immense implications for stem cell therapeutics in the context of precision medicine. AI/ML can be leveraged to identify common genomic traits, individual genetic polymorphism, disease-associated mutations, morphological patterns, and/or cellular functions in a personalized manner (Capponi and Daniels, 2023). This transdisciplinary knowledge helps: 1) determine the developmental stage and maturation of stem cells, (Guan et al., 2021; Kim et al., 2022) 2) assess their regenerative potentials and/or limitations, (Fischbacher et al., 2021), and 3) predict their therapeutic efficacy and/or side-effects in individual subjects. (Mota et al., 2021). For example, ML algorithms were used to identify biomarkers for predicting positive patient responses to BMSC therapy, (Steinhoff et al., 2017), characterize CMs non-invasively using video microscopy and image analysis, (Maddah and Loewke, 2014), analyze the effects of drugs on the calcium signals of iPSC-CMs, (Juhola et al., 2021), identify cell lines with/without genetic defects using cellular images, (Kim et al., 2023), and identify neural stem cell differentiation. (Zhu et al., 2021).

Moreover, by analyzing the unique DNA methylation profiles, investigators devised a linear classification learning model to discern iPSCs, ESCs, somatic cells, and embryonal carcinoma cells, achieving 94.23% accuracy. (Nishino et al., 2021). Another group utilized convolutional neural networks (CNNs) to effectively differentiate pluripotent cells from initial differentiating cells. (Waisman et al., 2019). The training of the CNN model involved the use of light microscopic images of PSCs captured at different intervals after the induction process, including mouse-embryonic cells being induced to epiblast-like cells. (Waisman et al., 2019). Notably, the results demonstrated CNN’s remarkable capability to distinguish between differentiated and undifferentiated cells with 99% accuracy.

Importantly, AI can leverage information from separate studies, extensive datasets, and stem cell biobanks to create models that predict the outcomes of stem cell therapy for specific disease states. These models can potentially be applied to enhance stem cell proliferation, optimize their functions in the host environment, and/or predict the most effective population(s) for individuals with specific phenotypes of cardiomyopathy (Capponi and Daniels, 2023). Thus, integrating AI/ML into stem cell research holds great promise for advancing precursor cell-based therapy for HF by: 1) facilitating our understanding of stem cell biology within specific cardiac disease contexts at a systems level; 2) improving the good manufacturing practice for clinical-grade cellular products; and 3) establishing personalized therapeutic prediction models for individual patients (Figure 1).

## Discussion

A considerable number of recent clinical trials in stem cell therapy for HF have demonstrated its promise and substantially increased our understanding of the behaviors and working mechanisms of stem/progenitor cells in patients (Table 1). (Hamshere et al., 2015; Martino et al., 2015; Perin et al., 2015; Choudry et al., 2016; Noiseux et al., 2016; Patel et al., 2016; Bartolucci et al., 2017; Butler et al., 2017; Choudhury et al., 2017; Florea et al., 2017; Gwizdala et al., 2017; Hare et al., 2017; Steinhoff et al., 2017; Teerlink et al., 2017; Xiao et al., 2017; Bassetti et al., 2018; Vrtovec et al., 2018; Yau et al., 2019; Bolli et al., 2020; He et al., 2020; Makkar et al., 2020; Mathiasen et al., 2020; Ulus et al., 2020; Bolli et al., 2021; Qayyum et al., 2023a; Qayyum et al., 2023b; Perin et al., 2023) In the next phase of clinical stem cell research, it is critical to address the outcome discrepancy between preclinical and clinical studies and expand the scope of stem cell-based therapy to other forms of cardiomyopathy, such as chemotherapy- or arrhythmia-induced cardiomyopathy. Exploiting the power of AI/ML and computational tools will facilitate our understanding of the benefits and limitations of stem cell therapy and provide a systems perspective for properly applying stem cell therapeutics in the context of precision and personalized medicine.
